# Supplementary material for: The Construction and Exploration of a Comprehensive MicroRNA Centered Regulatory Network in Foxtail Millet (Setaria italica L.)
Source: Front Plant Sci. 2022 May 6;13:848474. doi: 10.3389/fpls.2022.848474 (PMC9121102; doi:10.3389/fpls.2022.848474)
Supplement: Supplementary file 7 [file Data_Sheet_7.zip › Sit-miRNovel2_Seita.7G125300.1_225_TPlot.pdf]

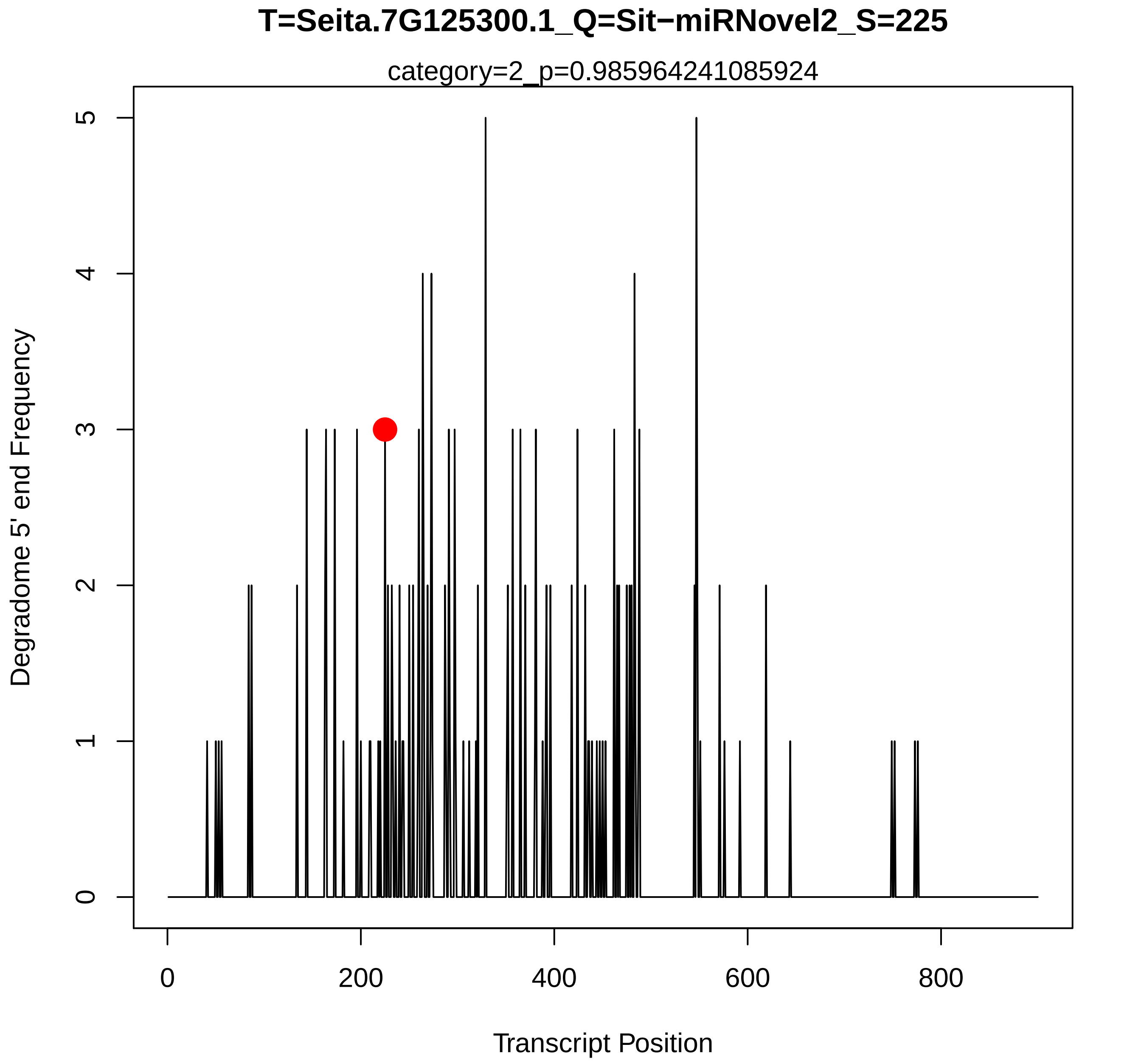

T=Seita.7G125300.1_Q=Sit−miRNovel2_S=225
category=2_p=0.985964241085924
Degradome 5' end Frequency
0
1
2
3
4
5
0
200
400
600
800
Transcript Position
